# Effects of Macronutrients in Ten Different Plant Species on the Food Choice and Growth Performance of *Achatina fulica*

**DOI:** 10.3390/ani15223333

**Published:** 2025-11-19

**Authors:** Kelin Tang, Zuohang Zheng, Xi Lu, Zhiqiang Han, Shaolei Sun

**Affiliations:** Fishery College, Zhejiang Ocean University, Zhoushan 316022, China; donkelin994@163.com (K.T.); zzh031214lhm@163.com (Z.Z.); 17280539209@163.com (X.L.); d6339124@163.com (Z.H.)

**Keywords:** *Achatina fulica*, feeding preference, growth performance, macronutrients

## Abstract

The giant African snail (*Achatina fulica*), a globally invasive mollusk, poses a serious threat to agricultural production and the ecological environment. However, few studies have focused on the feeding preference and growth performance of *A. fulica* on different plants, with especially few reports on the correlation between the nutrient content of plants and its growth performance. In this study, 10 plant species, including lettuce, stem lettuce, spinach, Chinese cabbage, cabbage, rape, apple, pear, banana, and pitaya, were selected as food sources to explore their effects on the feeding and growth performance of *A. fulica*. The results showed that *A. fulica* had the highest selection rate for lettuce and the lowest for rape. Feeding consumption (FC), daily body growth (DBG), daily increase in shell diameter (DISD), daily increase in shell length (DISL), relative consumption rate (RCR), and relative growth rate (RGR) of snails feeding on lettuce were significantly higher than those in the other nine groups. FC, DBG, DISD, DISL, RCR, and RGR were significantly correlated with the nutrient contents (protein, carbohydrate) of the 10 plants and with the protein-to-carbohydrate (P:C) ratio. Finally, based on the correlation between the macronutrient content of plants and the growth performance of *A. fulica*, we determined that these snails exhibit the optimal growth performance when fed food with the relative balanced P:C ratios (0.41–0.66) while having poor growth performance when fed plants with extremely imbalanced P:C ratios (rape: 2.45 or fruits: 0.04–0.13). Our study shows that *A. fulica* may cause potential economic losses for many cultivated plants, particularly lettuce, and provides a foundation for certain research values for agricultural prevention and ecological environment protection.

## Simple Summary

The giant African snail (*Achatina fulica*) is globally recognized as one of the most invasive mollusk species and is identified as a serious threat to agricultural production and the ecological environment. Although previous studies have demonstrated the preferential feeding habits, growth, and reproduction performance of *A. fulica* on several plant species, few studies have focused on the correlation between the nutrient content of plant species and its growth performance. Therefore, the present study first selected 10 different plant species as food sources for it and investigated the effects of these plants on its feeding preference and growth performance. Secondly, the correlations between plant nutritional components and its growth performance were analyzed. The results showed that plants’ nutrient content had a significant effect on the feeding preference and growth performance of *A. fulica*. Based on these findings, our study identifies a rough optimal nutritional intake range for *A. fulica* when fed different plant species.

## 1. Introduction

The giant African snail (*Achatina fulica*), a terrestrial gastropod of the family *Achatinidae*, is recognized as one of the most invasive mollusk species in the world. It has been included in the list of 100 high-profile invasive species that was formulated by the International Union for Conservation of Nature (IUCN) [1]. Furthermore, the giant African snail was recently listed in China’s First Catalog of Key Managed Invasive Alien Species in 2023. The giant African snail has rapidly spread throughout East Africa, South America, and Asia [2,3]. In China, it has widely expanded to many southern provinces, causing severe damage to agricultural crops and horticultural plants [4]. Therefore, it is considered a serious threat to crop and horticultural production.

Annually, invasive species cause great threats to ecological security and agricultural productivity, while concurrently resulting in substantial economic losses [5]. Invasive species generally have a strong adaptability to environments. Climate change is an important factor influencing the distribution of invasive species, and there have been numerous studies focusing on predicting the potential distribution areas of invasive species based on climate factors [6]. Furthermore, invasive animals possess a strong trophic plasticity [7,8]. However, few studies have reported on predicting the distribution range of invasive species based on their specific nutritional adaptation ranges. Under natural conditions, terrestrial invasive animals can feed on many plant species. However, the quality of different plant species, such as the levels of nutrients, has an important impact on the feeding selectivity and growth performance of terrestrial invasive animals [9]. Similar to other terrestrial invasive animals, *A. fulica* encounters heterogeneous nutritional environments in nature, and dietary quality may critically regulate its feeding selectivity and growth performance. Previous studies indicate that, compared with native gastropods, this invasive species exhibits strong ecological plasticity, surviving across broader nutritional ranges [10,11,12]. How does *A. fulica* regulate its feeding in a heterogeneous nutritional environment? It would be valuable to explore this question in depth. Hence, understanding the effects of specific nutritional components in host plants on the food choice and growth performance of *A. fulica* fundamental data for the future establishes a nutrition-based ecological niche model to predict the potential distribution areas of this snail and enables more effective monitoring and management of its spread.

In recent years, there have been numerous studies investigating the effects of different host plants and nutrients on the growth and development performance of invasive species [13,14,15,16]. For example, research on *Spodoptera frugiperda* revealed that variations in host plants and the levels of nutrients among host plants critically shape its survival strategies, with significant differences in host-specific oviposition selection, larval feeding rates, and growth performance of *S. frugiperda* observed when fed different host plants [9]. However, studies on the invasive snails mainly investigated how different plant species and artificial diet affect its growth and development, and studies on the feeding and growth preferences of snails in relation to the nutrient composition of plants are scarce. For example, when fed three leafy vegetables, *A. fulica* performed better weight gain than when fed the fluted pumpkin leaf, while the result obtained recorded no significant differences [17]. Furthermore, when fed different artificial diets, the *Achatina achatina* had the best growth performance under a low energy level diet [18]. These studies all indicated that different nutritional statuses significantly affect the growth performance of invasive species.

It is well known that all animals require sufficient nutrients from their diets to maintain their life history, such as growth and reproduction [19,20]. Macronutrients, such as proteins and carbohydrates, are fundamental nutritional elements in host plants and are crucial for animal growth and development. Proteins contribute to the formation of tissues, cells, and organ systems, while carbohydrates provide energy substances for the growth and development of animals [21,22]. Therefore, investigating the relationship between these two foundational nutrients in host plants and the feeding selectivity and growth performance of *A. fulica* is of paramount importance. Furthermore, to clarify the optimal protein to carbohydrate ratio for *A. fulica* when feeding on host plants can provide reference data to design artificial diets for *A. fulica* and also contributes to a further understanding of the behavioral and physiological basis of the snail’s response to different nutritional statuses.

To date, the growth and reproduction performance of *A. fulica* fed different plants has been investigated [23]. However, few studies focus on the relevance of protein and carbohydrate composition in host plants for the feeding and growth performance of *A. fulica*. Hence, in this study, we first investigated the feeding selectivity of *A. fulica* regarding 10 plant species, i.e., lettuce, stem lettuce, spinach, Chinese cabbage, cabbage, rape, apple, pear, banana, and pitaya. Second, the food consumption and growth performance of *A. fulica* fed the 10 plant species were studied. Finally, the protein and carbohydrate contents of 10 plant species were detected, and the correlation between the protein/carbohydrate contents and growth performance of *A. fulica* was analyzed. This study will help us to understand the potential damage caused by *A. fulica* to different plant species, clarify the effects of proteins and carbohydrates in these plants on their growth indices, and provide new insights into elucidating the underlying mechanisms behind the strong nutritional adaptability of *A. fulica* as an invasive species.

## 2. Materials and Methods

### 2.1. Snail Rearing and Plant Species

The giant African snails were purchased from Yimu Farm Co., Ltd. in Luoyang City, Henan Province, China. All snails were reared separately in clear plastic containers in a climate room (22 ± 1 °C, 55% RH, and 14 L:10 D photoperiod). Prior to the start of all the experiments, snails were starved for two days. Then, one-month-old snails with a shell length of 2–3 cm and a weight of 1.97 ± 0.42 g were selected for subsequent experiments.

Ten different plant species, i.e., the leaves of lettuce (*Lactuca sativa* var. *ramosa* Hort.) (Poales: Asteraceae), stem lettuce (*Lactuca sativa* var. *angustana*) (Poales: Asteraceae), spinach (*Spinacia oleracea*) (Poales: Amaranthaceae), Chinese cabbage (*Brassica pekinensis*) (Poales: Brassicaceae), cabbage (*Brassica oleracea*) (Poales: Brassicaceae), and rape (*Brassica napus*) (Poales: Brassicaceae) and the fruits of apple (*Malus domestica*) (Poales: Rosaceae), pear (*Pyrus communis*) (Poales: Rosaceae), banana (*Musa acuminata*) (Poales: Musaceae), and pitaya (*Hylocereus undatus*) (Poales: Cactaceae) were selected to test the feeding selectivity and growth performance of snails.

### 2.2. Feeding Selectivity of Snails Regarding 10 Plant Species

**Figure 1:**
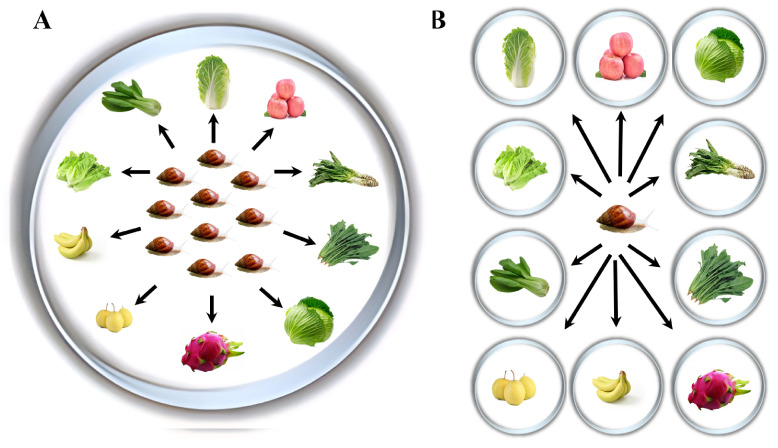
The design of choice experiment (**A**) and non-choice experiment (**B**). Prior to the start of all the experiments, snails were starved for two days. In the choice experiment, three replicates were established, each replicating 10 snails and 10 different plants. In the no-choice experiment, 10 treatments were designed and each treatment had 10 replicates. Ten different plants represented one treatment. For each treatment, sufficient food was provided to the snails to ensure there was surplus after the snails’ feeding.

In the first experiment (choice experiment), the one-month-old snails were randomly assigned to three rearing boxes (50 cm in diameter and 20 cm in height) containing 10 plant species, i.e., three replicate groups of 10 giant African snails each, as shown in Figure 1A. During the experiment, the number of giant African snails on each of the 10 plants was recorded 3 times a day (at 8:00, 12:00, and 16:00) and lasted for 10 days. The plants were replaced every day to ensure the freshness of the plants. Each time, sufficient food was fed to guarantee that after feeding plants were surplus. The remaining plants were collected and weighed to calculate the food consumption of the snails. Finally, based on the number of giant African snails on different plants and the food consumption of the snails, the feeding selective preference of giant African snails was determined.

### 2.3. Feeding and Growth Performance of Snails

In the second experiment (no-choice experiment), the one-month-old snails were weighed and individually transferred into a container (5 cm in diameter and 3 cm in height) and 1 of the 10 plant species was given (Figure 1B). Each treatment had 10 replicates. During the experimental period, plants were changed every day to ensure freshness. For each treatment, sufficient food was provided for the snails, and the mass of the plants was measured before and after 24 h of feeding. The food consumption of snails was calculated according to original feeding minus remaining plants after 24 h. Furthermore, the growth performance, including shell length, shell diameter, and weight of giant African snails were measured before and after 15 days of feeding.

Finally, feeding consumption (FC), daily body growth (DBG), daily increase in shell length (DISL), daily increase in shell diameter (DISD) [24], relative consumption rate (RCR), and relative growth rate (RGR) were calculated by using the following formulae [25]:

FC = (A − B)/T

DBG = (D − C)/T

DISL = (F − E)/T

DISD = (H − G)/T

$$\mathrm{RCR}=\frac{2(A-B)}{(C+D)/T}$$

$$\mathrm{RGR}=\frac{2(D-C)}{(C+D)/T}$$

where A = pre-feeding weight of plant, B = post-feeding weight of plant, C = initial weight of snail, D = final weight of snail, E = initial length of the snail shell, F = final length of the snail shell, G = initial diameter of the snail shell, H = final diameter of the snail shell, and T = experiment periods.

### 2.4. Analysis of Protein and Carbohydrate Contents of 10 Plant Species

The digestible carbohydrate (soluble sugar) content in plant samples was determined by the anthrone colorimetric method [26]. Approximately 0.5 g of dried plant material was ground and extracted with 10 mL of 80% (*v*/*v*) ethanol at 80 °C for 40 min in a water bath. After cooling to room temperature, the homogenate was centrifuged at 4000× *g* for 10 min, and the supernatant was retained. The extraction procedure was repeated twice. The combined supernatants were transferred to a 50 mL volumetric flask and brought to volume with 80% ethanol. For quantification, 0.2 mL of the extract was mixed with 0.8 mL distilled water and 4 mL anthrone reagent, followed by heating in a boiling water bath for 10 min. After cooling, the absorbance was measured at 630 nm using a microplate reader (Infinite M200, Tecan Trading AG, Männedorf, Switzerland). A standard curve was prepared using D-glucose (Guangdong Guanghua SciTech Co., Ltd., Shantou, Guangdong, China) as reference. For each species, 15 biological replicates were analyzed, with each replicate consisting of 5 individual plants pooled from 3 independent experiments.

Soluble protein content was assessed using the Bradford method [27]. Approximately 1 g of fresh plant tissue was homogenized in 5 mL of distilled water in an ice bath and centrifuged at 4000× *g* for 10 min. The supernatant was collected, and the process repeated twice. Then, 0.1 mL of the supernatant was reacted with 5 mL of Coomassie Brilliant Blue G-250 (Shanghai Yuanye Bio-Technology Co., Ltd., Shanghai, China) reagent and 0.9 mL distilled water. Absorbance was read at 595 nm using a microplate reader (Infinite M200, Tecan Trading AG, Switzerland). Bovine serum albumin (BSA) (Beijing Solarbio Science & Technology Co., Ltd., Beijing, China) was used as the protein standard. Each plant sample was evaluated in 15 biological replicates, with 5 plants combined per replicate across 3 trials.

### 2.5. Data Analysis

The feed selection rate, feeding consumption, and growth performance of the giant African snails in the choice experiment and the non-choice experiment were analyzed by one-way analysis of variance (ANOVA), and significances between means were obtained by Tukey’s HSD test. Furthermore, the correlation between the nutrient content of plants and the growth performance of giant African snails was tested by simple linear regression. All analyses were conducted in Statistical Product and Service Solutions V.27.0 software (SPSS, Chicago, IL, USA), and figures were generated using GraphPad Prism 9.50 (GraphPad Software, San Diego, CA, USA).

## 3. Results

### 3.1. Feeding Selectivity of Snails Regarding 10 Plant Species in Choice Experiment

**Figure 2:**
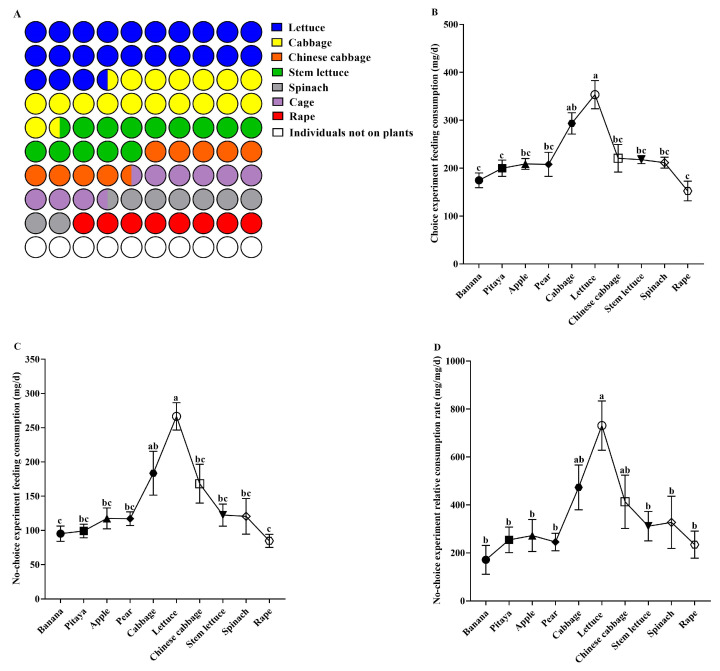
Feeding preference of the giant African snail. (**A**) Food selection rate of choice experiment; (**B**) feeding consumption of choice experiment (mean ± SE); (**C**) feeding consumption of no-choice experiment (mean ± SE); (**D**) relative consumption rate of no-choice experiment (mean ± SE). The lowercase letters (e.g., a, b, c, ab, bc) indicate significant differences in feeding preference among the treatment groups.

The feeding selectivity of giant African snails was affected by different plant species (Figure 2A, F = 26.082, df = 6, *p* < 0.001). The percentage of snails feeding on lettuce was the highest, accounting for 37.21%. However, no snails were observed on apple, pear, pitaya, or banana.

In the choice experiment, the giant African snails had the highest feeding consumption on lettuce (353.4 mg/d) and the lowest was on rape (152.4 mg/d) (Figure 2B, F = 8.290, df = 9, *p* < 0.001).

### 3.2. Feeding and Growth Performance of Snails in No-Choice Experiment

In the no-choice experiment, the food consumption of snails to different plant species are presented in Figure 2C,D. From these results, it is clear that the different plant species significantly affected the snails’ food consumption (FC: F = 7.909, df = 9, *p* < 0.001; RCR: F = 4.211, df = 9, *p* < 0.05). The snails had the highest food consumption (266.6 mg/d) and relative consumption rate (730 mg/mg/d) for lettuce, consistent with the choice experiment.

**Figure 3:**
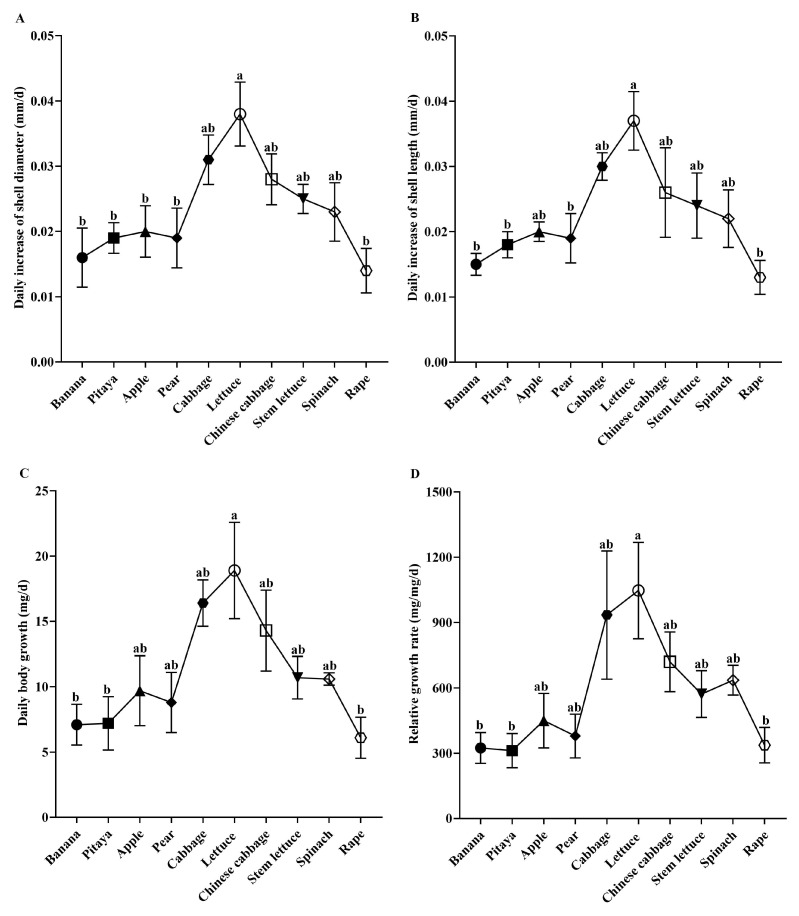
Growth performance variations in the giant African snail. (**A**) Daily increase in shell diameter (DISD) (mean ± SE); (**B**) daily increase in shell length (DISL) (mean ± SE); (**C**) daily body growth (DBG) (mean ± SE); (**D**) relative growth rate (RGR) (mean ± SE). The lowercase letters (e.g., a, b, ab) indicate significant differences in growth performance among the treatment groups.

The growth performance of daily increase in shell length (DISL), daily increase in shell diameter (DISD), daily body growth (DBG), and relative growth rate (RGR) of snails varied significantly among ten plant species (Figure 3, DBG: F = 3.568, df = 9, *p* < 0.001; DISL: F = 3.544, df = 9, *p* < 0.001; DISD: F = 3.555, df = 9, *p* < 0.001; RGR: F = 3.214, df = 9, *p* < 0.05). Four growth performances were all the highest when snails were fed lettuce, followed by cabbage, Chinese cabbage, stem lettuce, spinach, apple, pear, pitaya, banana, and rape.

### 3.3. Protein and Carbohydrate Contents of 10 Plant Species

**Figure 4:**
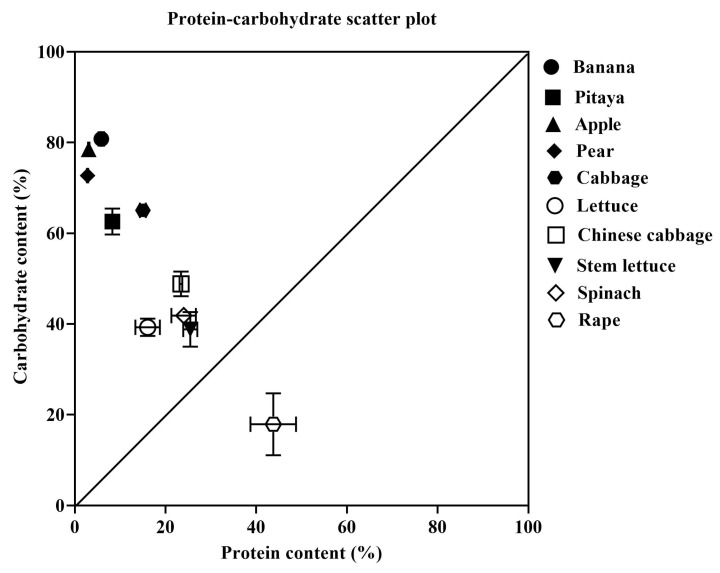
The protein/carbohydrate scatter plot of 10 different plants.

The protein and carbohydrate contents of ten plant species varied significantly (Figure 4). Banana had the highest carbohydrate content, followed by apple, pear, cabbage, pitaya, Chinese cabbage, spinach, lettuce, stem lettuce, and rape. The protein content of rape was the highest, followed by stem lettuce, spinach, Chinese cabbage, lettuce, cabbage, pitaya, banana, apple, and pear. Furthermore, the protein (P)/carbohydrate (C) ratio of 10 plants were calculated, i.e., lettuce (P:C = 0.41), cabbage (P:C = 0.23), Chinese cabbage (P:C = 0.48), stem lettuce (P:C = 0.66), spinach (P:C = 0.57), apple (P:C = 0.04), pear (P:C = 0.04), banana (P:C = 0.07), pitaya (P:C = 0.13), and rape (P:C = 2.45).

### 3.4. Correlation Between Protein/Carbohydrate Contents of 10 Plant Species and Growth Performance of the Giant African Snails

**Figure 5:**
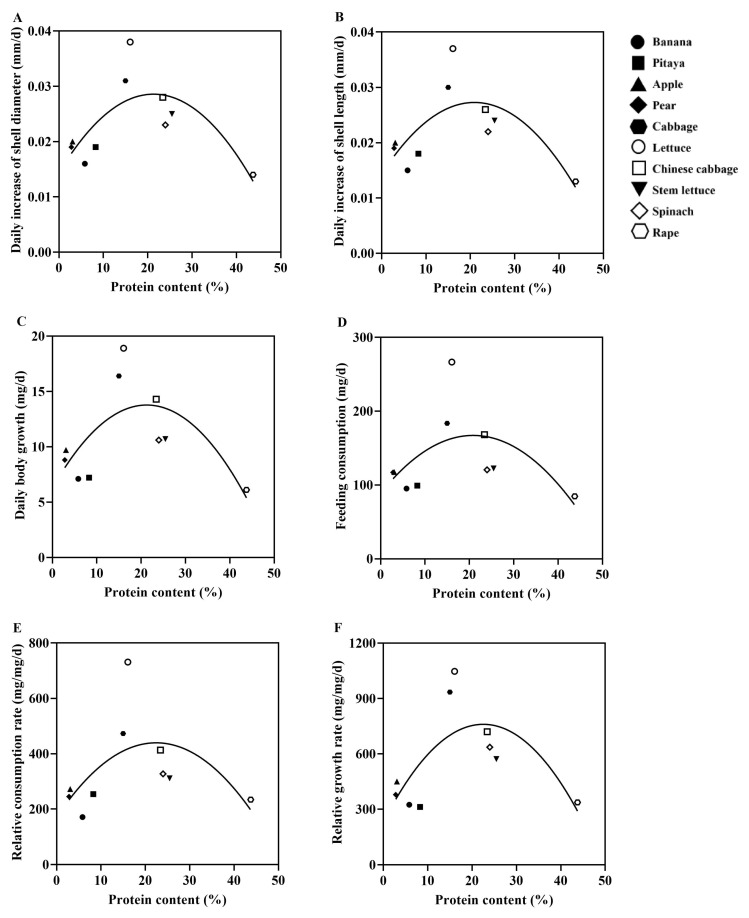
Correlation between plant protein content and growth performance of the giant African snails in non-choice experiments. Each different graphic represents a different plant species. Simple linear regression was used to fit the relationship between the protein content of ten different plant species and the growth performance of the snails. (**A**) Daily increase in shell diameter (DISD); (**B**) daily increase in shell length (DISL); (**C**) daily body growth (DBG); (**D**) feeding consumption (FC); (**E**) relative consumption rate (RCR) (**F**) relative growth rate (RGR).

**Figure 6:**
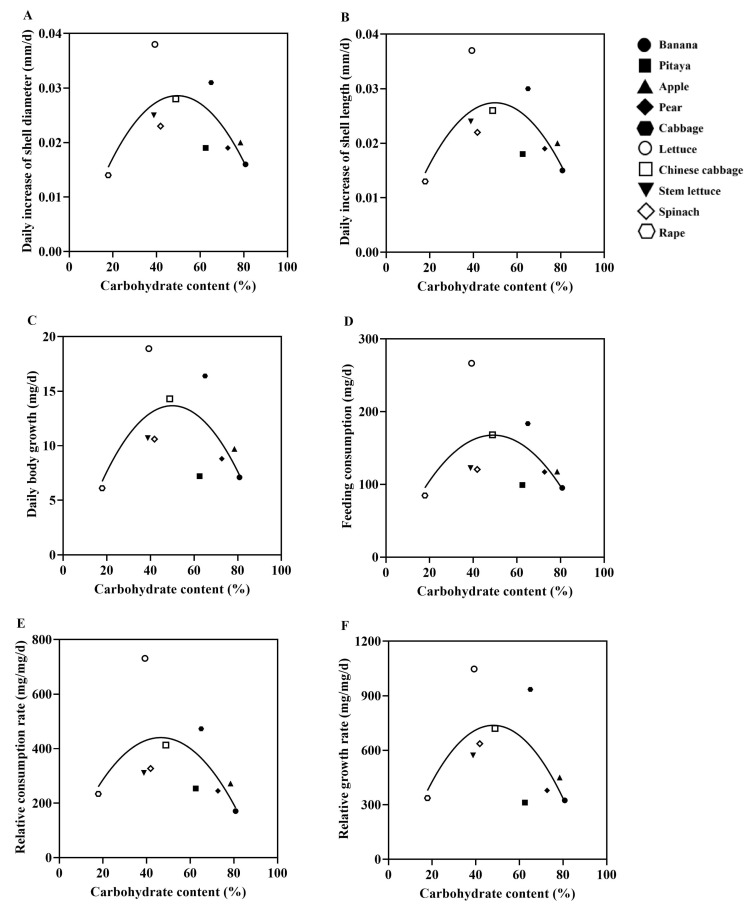
Correlation between plant carbohydrate content and growth performance of the giant African snails in non-choice experiments. Each graphic represents a different plant species. Simple linear regression was used to fit the relationship between the carbohydrate content of ten different plant species and the growth performance of the snails. (**A**) Daily increase in shell diameter (DISD); (**B**) daily increase in shell length (DISL); (**C**) daily body growth (DBG); (**D**) feeding consumption (FC); (**E**) relative consumption rate (RCR); (**F**) relative growth rate (RGR).

Based on the results of the no-choice experiment, simple linear regression analysis revealed a significant correlation between the protein/carbohydrate contents of plants and the growth performance of the snails (Figure 5 and Figure 6). The giant African snails exhibited the best growth performance when feeding on lettuce (P:16.07%, C:39.27%), followed by those feeding on cabbage (P:15.00%, C:65.03%).

## 4. Discussion

This study provides a nutrient-level mechanistic explanation for the feeding ecology of *A. fulica*. Results indicate that different plant species significantly influence the feeding preference and growth performance of *A. fulica*. We found that *A. fulica* exhibited a strong feeding preference for lettuce (P:C = 0.41), followed by cabbage (P:C = 0.23), Chinese cabbage (P:C = 0.48), stem lettuce (P:C = 0.66), and spinach (P:C = 0.57). This strong preference was not arbitrary but was significantly correlated with the protein and carbohydrate contents of the plants, pointing towards an underlying nutritional regulatory mechanism driving the snails’ foraging behavior and growth performance. Moreover, previous studies have demonstrated that *Archachatina marginata* prefers wild plants with a high protein and lipid content, and incorporating 18% crude protein into the diets can significantly improve the growth performance of *A. fulica* [20,28]. This finding aligns with the principles of the geometric framework for nutrition, which posits that animals regulate their intake of nutrients to reach an optimal growth performance [29]. Additionally, this ability to adjust feeding intake to reach an optimal growth performance is widely present among animals, such as insects, mammals, and marine species [16,30,31].

*A*. *fulica* had poor performance and low feeding selection when fed plants like rape and fruits (apple, pear, banana, pitaya). These results can be explained by the nutrient level of these plants’ deviation from this optimal nutrient profile [32,33,34]. Rape, despite its high protein content, possesses an extremely high P:C ratio (2.45), which likely creates a nutritional imbalance, potentially leading to metabolic inefficiencies or toxicity from excess protein if consumed in large quantities [35]. In contrast, the fruits exhibited very low P:C ratios (0.04–0.13), being extremely carbohydrate-rich and protein-poor. While they may provide enough energy, they lack sufficient protein to support the structural growth and developmental needs of the snails [36]. This was consistent with previous studies on the feeding habits of snails; when nutrients in the natural environment fail to meet their normal requirements, the snails will enter a dormant state and exhibit low growth performance [37]. These results highlight the importance of nutrient balancing for this species.

As we all know, the nutrient content of plants is one of the major factors influencing the feeding preference and growth performance of animals [38,39]. In the present study, we found that *A*. *fulica* exhibited optimal feeding preference and growth performance when fed plants with relatively balanced contents of protein and carbohydrate, while exhibiting low feeding preference and growth performance for plants with imbalanced contents of protein and carbohydrate. Similar to a previous study, the invasive species *S. frugiperda* only exhibited optimal growth performance when they fed on nutritionally balanced food [9]. Interestingly, our results differ with previous findings in snail species when fed other plant species. For instance, Jimoh et al. found that *A. marginata* exhibited optimal growth performance on diets containing *Moringa oleifera* and *Leucaena leucocephala* leaf, which have high protein levels [40]. Furthermore, Oyeagu et al. reported that, when fed the *Centrosema pubescens* leaf, a legume with high protein quality, *A. marginata* exhibited the best growth and reproductive traits [41]. These studies collectively underscore the importance of dietary protein for snail growth. However, our research demonstrated that protein is not the sole determinant, carbohydrates are equally critical. This explains some other studies that found no significant differences in the growth performance of *A. fulica* when fed three different leafy vegetables (*Vernonia amygdalina*, *Telfairia occidentalis*, *Carica papaya*), which, due to the P:C ratios of these plants, did not deviate from the snail’s intake target in the results in our study.

While this study firmly establishes the role of two macronutrient (proteins and carbohydrates) contents of plants for snails’ growth performance, the micronutrient contents (especially calcium for shell formation) of plants, which were not measured here, also influence the growth performance of snails [42]. Previous studies show that dietary calcium exhibits significant effects on the growth, development, reproduction, and shell formation of snails [43]. Hence, future studies should incorporate analyses of these additional compounds and use more detailed nutritional geometry approaches to create a multi-dimensional nutrient intake landscape for *A. fulica*.

In total, for most animals, foraging in complex and variable natural environments poses a challenge [44,45]. When dietary nutrients are inadequate, animals usually adjust their food intake to compensate for nutritional deficits [46]. In this study, we found that *A. fulica* actively adjusted food intake; however, under these conditions, *A. fulica* did not fully achieve optimal growth performance. Ultimately, our study identifies a rough optimal nutritional intake range for *A. fulica* when fed different plant species. The practical implications of these findings are twofold. First, our results provide fundamental data for the future to establish a nutrition-based ecological niche model to predict the potential distribution areas of this snail and enable more effective monitoring and management of its spread. Second, our results can provide reference data to design artificial diets for *A. fulica* and contribute to a further understanding of the behavioral and physiological basis of the snail’s response to different nutritional statuses using the principles of the geometric framework for nutrition.

## 5. Conclusions

In this study, we first identified that *A. fulica* exhibits the optimal feeding preference for lettuce, followed by cabbage and other plant species. Secondly, based on the correlation between the macronutrient content of plants and the growth performance of *A. fulica*, we determined that these snails exhibit optimal growth performance when fed food with the relative balanced P:C ratios (0.41–0.66), while they have poor growth performance when fed plants with extremely imbalanced P:C ratios (rape: 2.45 or fruits: 0.04–0.13). Our results provide fundamental data for establishing a nutritional niche prediction model and the development of an artificial diet for *A. fulica*. Furthermore, according to this conclusion, the corresponding preventive measures for *A. fulica* can be carried out to protect economic crops and the ecological environment.

## Data Availability

The data presented in this study are available from the corresponding author upon reasonable request.
